# Diversity and Metabolism of Microbial Communities in a Hypersaline Lake along a Geochemical Gradient

**DOI:** 10.3390/biology11040605

**Published:** 2022-04-15

**Authors:** Alla V. Bryanskaya, Aleksandra A. Shipova, Alexei S. Rozanov, Oxana A. Kolpakova, Elena V. Lazareva, Yulia E. Uvarova, Vadim M. Efimov, Sergey M. Zhmodik, Oxana P. Taran, Tatyana N. Goryachkovskaya, Sergey E. Peltek

**Affiliations:** 1 Laboratory of Molecular Biotechnologies, Federal Research Center Institute of Cytology and Genetics SB RAS, 630090 Novosibirsk, Russia; aleksa120396@yandex.ru (A.A.S.); rozanov@bionet.nsc.ru (A.S.R.); ov@bionet.nsc.ru (O.A.K.); elfa1986@mail.ru (Y.E.U.); efimov@bionet.nsc.ru (V.M.E.); tanago@bionet.nsc.ru (T.N.G.); peltek@bionet.nsc.ru (S.E.P.); 2 Kurchatov Genomics Center, Federal Research Center Institute of Cytology and Genetics SB RAS, 630090 Novosibirsk, Russia; 3 V.S. Sobolev Institute of Geology and Mineralogy SB RAS, 630090 Novosibirsk, Russia; lazarevael@mail.ru (E.V.L.); zhmodik@igm.nsc.ru (S.M.Z.); 4 FRC Krasnoyarsk Science Center SB RAS, Institute of Chemistry and Chemical Technology SB RAS, 660036 Krasnoyarsk, Russia; oxanap@catalysis.ru

**Keywords:** metagenome, saline lake, microbial community, metabolic pathway, biogeochemical cycle

## Abstract

**Simple Summary:**

In the south of western Siberia (Russia), there are many unique and unexplored lakes. This work focused on Lake Solenoe, a component of a poorly studied lake system in the south of western Siberia. The diversity of microorganisms of Lake Solenoe was evaluated in the floating community and in the bottom sediments. The aim was to assess the metabolic potential of this lake ecosystem and to describe the identified relations between the mineral and living components. This is the first detailed study on the microbial diversity, its metabolic potential, and their relations with a series of geochemical parameters in the lake ecosystem of Novosibirsk Oblast.

**Abstract:**

In the south of western Siberia (Russia), there are many unique and unexplored soda, saline, and freshwater lakes. In this study, the results are presented on microbial diversity, its metabolic potential, and their relation with a set of geochemical parameters for a hypersaline lake ecosystem in the Novosibirsk region (Oblast). The metagenomic approach used in this work allowed us to determine the composition and structure of a floating microbial community, the upper layer of silt, and the strata of bottom sediments in a natural saline lake via two bioinformatic approaches, whose results are in good agreement with each other. In the floating microbial community and in the upper layers of the bottom sediment, bacteria of the Proteobacteria (Gammaproteobacteria), Cyanobacteria, and Bacteroidetes phyla were found to predominate. The lower layers were dominated by Proteobacteria (mainly Deltaproteobacteria), Gemmatimonadetes, Firmicutes, and Archaea. Metabolic pathways were reconstructed to investigate the metabolic potential of the microbial communities and other hypothetical roles of the microbial communities in the biogeochemical cycle. Relations between different taxa of microorganisms were identified, as was their potential role in biogeochemical transformations of C, N, and S in a comparative structural analysis that included various ecological niches.

## 1. Introduction

Soda and saline lakes are inhabited by diverse communities of haloalkaliphilic microorganisms, mainly prokaryotes [1,2,3], which are well adapted to survival and growth under these extreme conditions and constitute similar functional microbial groups among such lakes worldwide [4,5,6,7,8,9]. Temperature, total organic carbon, K^+^, and Mg^2+^ are critical factors for the formation of the taxonomic structure of microbial communities in extremely saline ecosystems [10]. In this context, the relative abundance of various microbial groups is usually determined by brine salinity [11]. In soda lakes with high salinity (>5% *w/v*), complete biogeochemical cycles of carbon, nitrogen, and sulfur are in operation. Microbiological nutrient cycles are partially impeded only under the conditions of salt saturation. It is thought that moderately salinized soda lakes are among the most productive aquatic environments on Earth [12].

The investigation into unique extremophilic microbial communities inhabiting soda and saline lakes has revealed the presence of most of the functional microbial groups involved in the cycles of carbon, nitrogen, sulfur, and other elements [13]. For example, it is known that the layers of weakly alkaline ecosystems with high salinity that are close to the bottom are characterized by a developed cycle of sulfur dissimilation, which is implemented by a large number of taxonomic groups that are mostly unculturable under laboratory conditions [9].

The present work is focused on Lake Solenoe: a component of the poorly studied lake system in the south of western Siberia [14,15]. In the present study, we investigated the diversity of microorganisms of Lake Solenoe in the floating microbial community, in the upper layer of silt, and in the strata of bottom sediments by metagenomic techniques. Here, using the next-generation sequencing technology, by means of data on the taxonomic composition and on the sequences of genes and enzymes found in this community, we aimed to assess the metabolic potential of this lake ecosystem and to describe the identified relations between the mineral and living components. This is the first detailed study of the microbial diversity, its metabolic potential, and their relations with a series of geochemical parameters in the lake ecosystem of Novosibirsk Oblast.

The research into biodiversity in the saline lakes of the west Siberian lowland and into the processes of concentration of chemical microelements by microorganisms is of considerable interest, in part because bodies of water such as Lake Solenoe reflect the final stage in the lifespan of a sea basin. In fact, it is possible to investigate the processes associated with specific features of microbial communities and of their functioning including the accumulation of elements such as uranium, molybdenum, vanadium, arsenic, silver, and gold at the extraordinary final stage of sea basin degradation.

## 2. Materials and Methods

### 2.1. Sampling and DNA Extraction and Sequencing

In July 2017, on the coastal strip of Lake Solenoe, samples of water, microbial coating, and bottom sediments were collected for subsequent DNA extraction and metagenomic sequencing on the NextSeq 550 and NovaSeq 6000 platforms (Illumina, Inc., San Diego, CA, USA) [16] as well as for determining a set of geochemical parameters.

**Figure 1:**
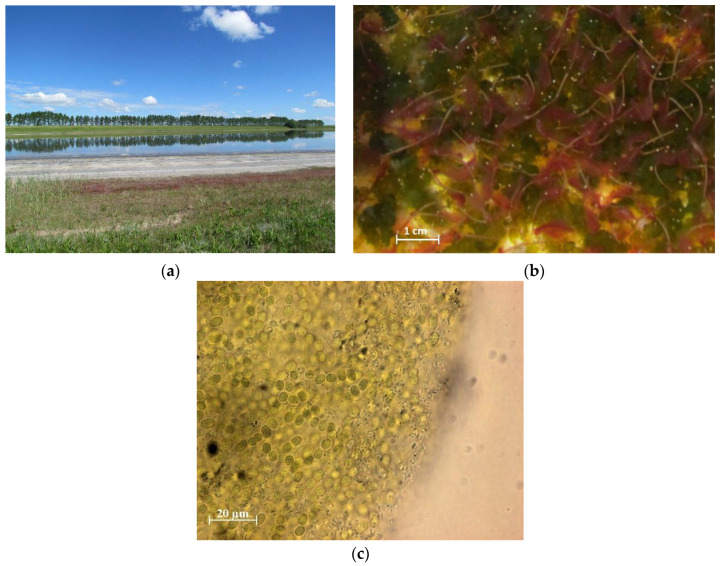
The coastal strip of Lake Solenoe (**a**). Loose cyanobacterial mass among *Artemia* sp. individuals (**b**). A colony of cyanobacteria from the R1 layer (**c**).

**Figure 2:**
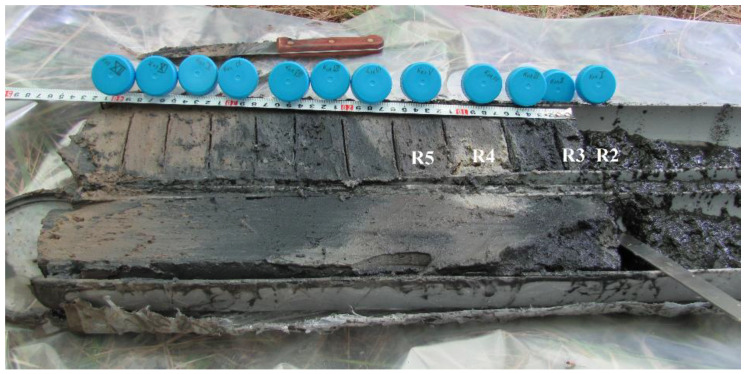
The column (core sample) of bottom sediments from Lake Solenoe (layers used for DNA isolation and metagenomic sequencing are marked in white).

Lake Solenoe is located near the village of Lepokurovo (Bagansky District, Novosibirsk Oblast) (54°11′57.44″ N, 78°10′4.83″ E). The study area has previously been described in detail [14,15]. Lake Solenoe has the following features that make it worthy of research: (1) the presence of annually developing cyanobacterial communities (mats or conglomerates, depending on the salinity of the environment; Figure 1); (2) the presence of thick strata of bottom sediments; (3) a set of geochemical parameters allowing for the detection and study of microorganisms with a wide range of ecophysiological and biochemical properties: polyextremophiles; (4) intensive development of the crustacean *Artemia*, which has special value and prospects for practical use (Figure 1); and (5) constant water abundance (high water cut): the lake never goes dry but is shallow enough for its level to change considerably, which makes it possible to track temporal changes in biological and geochemical characteristics.

In summer, floating microbial communities form on the coastal strip of the lake. In some years, they have substantial density and thickness (up to 6 cm), but in 2017, they looked like separate small (up to 1 cm) clusters of colonies (conglomerates) of cyanobacteria, bacteria, and algae.

Fifteen samples were obtained in this study for the “Collection of Biotechnological Microorganisms as a Source of Novel Promising Objects for Biotechnology and Bioengineering” at the Federal Research Center Institute of Cytology and Genetics, the Siberian Branch of the Russian Academy of Sciences (ICG SB RAS, Novosibirsk, Russia). The samples and layers were labeled as follows: R1, a floating microbial community; R2, the unconsolidated layer of a bottom sediment located at a depth of 0 to 3 cm; R3, the layer of a bottom sediment at 3 to 5 cm depth; R4, the layer of a bottom sediment at a depth of 10 to 15 cm; and R5, the layer of a bottom sediment at 15 to 19 cm depth (Figure 2). Three samples were collected from each studied layer. These samples were mixed, and an aliquot was taken from them for metagenomic analysis.

The samples of the floating microbial community and bottom sediments from the lake were placed into sterile 50 mL tubes and fixed by the addition of an equal volume of 96% ethanol. The samples were stored at −70 °C in the above-mentioned collection at the ICG SB RAS. Isolation of total DNA from the samples of the floating microbial community and bottom sediments was carried out using the Genomic DNA from Soil NucleoSpin^®^ Soil Kit (Macherey–Nagel Gmbh & Co., Düren, Germany) in accordance with the protocol recommended by the manufacturer. Isolation of total DNA from samples of the deeper layers of bottom sediments was performed as follows: 300 µL of a sample was placed in a tube containing ceramic beads for the grinding of solid particles and was centrifuged for 1 min at 16,000× *g*. The supernatant was discarded. To the precipitate, 500 μL of a buffer (100 mM Tris-HCl, 100 mM EDTA, pH 8.0) was added, and the mixture was vortexed. Next, 100 μL of lysozyme (10 mg mL^−1^) and 10 μL of an RNase solution (20 mg mL^−1^) were added, then incubated at 37 °C for 25 min, with shaking every 5 min. After that, 10 μL of proteinase K (800 U mL^−1^) was introduced, and the tubes were incubated for 10 min at 65 °C, followed by the addition of 100 μL of 10% SDS, 100 μL of 10% sarcosyl (Sigma-Aldrich, Inc., St. Louis, MO, USA), and 100 μL of chloroform. The cycle was performed three times: freezing in liquid nitrogen, thawing at 65 °C, and vortexing. Next, the samples were vortexed for a minute and supplemented with 100 μL of 10% polyvinylpyrrolidone (Sigma), frozen in liquid nitrogen, thawed at 65 °C, and vortexed, followed by centrifugation at 13,000× *g* for 10 min. The supernatant was transferred to a sterile tube, and an equal volume of isopropanol was added, with incubation for 30 min at −20 °C. Following this, the samples were centrifuged at 16,000× *g* for 15 min, and the supernatant was discarded. After that, 300 μL of 75% distilled ethanol was added, and the mixture was vortexed and centrifuged; the supernatant was discarded. The procedure was repeated. The precipitate was air dried with the lid open. Then, 150 μL of a buffer (10 mM Tris-HCl, 10 mM EDTA) was added. After the precipitate dissolved, the DNA was purified on magnetic particles CleanMag DNA (Evrogen, Moscow, Russia), according to the standard protocol. Libraries for sequencing with an average read length of 600 bp were prepared at the Multi-Access Center “Genomic Research” of the ICG SB RAS by means of the NEBNext(R) Ultra™ DNA Library Prep Kit for Illumina^®^ (New England BioLabs, Ipswich, MA, USA). For the samples of the floating microbial community and of the upper layer of bottom sediments, paired-end sequencing was performed at CGRM Genetico (Moscow, Russia) using the NovaSeq 6000 S1 Reagent Kit (200 cycles) on a NovaSeq instrument: insert length was ~450 bp, and read length 100 bp. For the samples of the other layers of bottom sediments, paired-end sequencing was performed at the Multi-Access Center “Genomic Research” at the ICG SB RAS, using the NextSeq 500/550 Mid Output Kit v2 (300 Cycles) on the NextSeq instrument: insert length was ~450 bp, and read length 150 bp.

### 2.2. Bioinformatic Analysis

The quality of the obtained reads was verified using FastQC. Next, the data obtained on the NovaSeq device were processed in Trimmomatic software (version 0.36) [17] with the settings CROP:97 MINLEN:95; the data from NextSeq were processed with the settings CROP:149 TRAILING:20 MINLEN:100. The reads obtained for the R4 bottom sediment layer were preprocessed in the cutadapt software (version 2.7) [18] with the following settings: -nextseq-trim 20 m 100. The reads were cleaned up by mapping them onto the human genome in bowtie2 (version 2.3.5) [19]; the reads that could be mapped were discarded. The assembly of reads into contigs was conducted using metaSPAdes software (version 3.11.1) [20]. For taxonomic analysis of the microbial communities, phyloFlash (version 3.3b2) [21] was used with default settings. Because the sequencing was genome-wide, less than 1% of the metagenomic reads from each sample was assigned taxonomically; phyloFlash software and 16S rRNA (and 18S rRNA) gene sequences of various microorganisms were employed.

For automated phylogenetic and functional analyses of metagenomes, MG-RAST Web server v4.0.3 [22] was used. MG-RAST automatically performs quality control and nucleotide and amino acid sequence alignments with database accession numbers. This approach allows one to perform taxonomic identification, to detect metabolic pathways, and to conduct a comparative analysis of metagenomes. The data-processing pipeline of MG-RAST includes read quality control (Solex aQA, DRISEE, Bowtie2) and annotation (FragGeneScan with the search against the protein M5nr database, which integrates databases GenBank, SEED, IMG, UniProt, KEGG, and eggNOGs).

### 2.3. Geochemical Methods

In 2017, core samples of bottom sediments were collected by means of a plastic pipe with a diameter of 100 mm, which was sawed in half lengthwise beforehand, and after the removal of the sediment, it was opened on the spot (Figure 2).

Bottom sediments were sampled in a coastal location, at the site of active development of the cyanobacterial community. The sampled material was divided into layers (Figure 2). First, the samples were taken for the metagenomic analysis (see above). In the upper two (water-rich) layers, pH and Eh were measured in situ in the suspension. After the samples for metagenomic analyses were withdrawn, to the material extracted from the inner part of the sediment (except for the upper two layers), distilled water was added at a ratio of 7.5 mL of sediment to 5 mL of water; the water was additionally purified beforehand with a Milli-Q (Millipore, Merck KGaA, Darmstadt, Germany) fine water purification unit (with pH 7.3 and Eh ≈ 400 mV). In the resulting suspension, pH and Eh were measured. The remaining material was hermetically packed, and after three days under stationary conditions, a pore solution was squeezed out of it by means of a manual laboratory press (expeller) at 150 kbar. pH and Eh were next measured in the solution. The solution was centrifuged, and a portion of it together with the colloid was acidified with concentrated HNO_3_. The second portion of the solution was stored without acidification for assays of anions and carbon. Samples of pore solutions from layers 1 and 2 contained a visible brown colloid, indicating a high concentration of organic carbon (C_org_). Before analysis, these samples were pre-oxidized using HNO_3_ and H_2_O_2_ with heating under an infrared lamp in a closed volume. The other samples were analyzed without the pretreatment. Elements were quantitated by inductively coupled plasma mass spectrometry. The average salinity of the solution was ~120 g L^−1^ (i.e., 12%). Therefore, logically, it was necessary to dilute the original samples at least 200-fold. To quantitate C_org_ and total carbon (C_tot_) in the lake solutions, in expeller extracts from the microbial community, and in pore solutions from bottom sediments, the samples were passed by means of a syringe through a filter nozzle made of cellulose acetate with a pore diameter of 0.45 μm. To quantitate organic and total carbon, a Multi N/C 2100S analyzer (Analytik Jena AG, Jena, Germany) was utilized. The total C content was calculated from the amount of CO_2_ released after thermocatalytic oxidation of a sample in a quartz reactor in a stream of O_2_ (flow rate 160 mL min^−1^) at 950 °C. Platinum on an Al_2_O_3_ support served as a catalyst. Inorganic carbon (C_inorg_) concentration was calculated from the amount of CO_2_ released after a sample was treated with a 10% solution of H_3_PO_4_. CO_2_ concentration in the carrier gas flow was determined on a nondispersive infrared-radiation detector. Organic carbon was quantified as the difference between the amounts of total and inorganic C. The standard deviation of this assay was <5%. The range of determined total organic carbon (TOC) concentrations was 0.002–30,000 mg L^−1^. The inorganic carbon content was converted to HCO_3_^−^ content.

The anionic composition of water samples was investigated by capillary electrophoresis (Kapel 103R, Lumex, operator: E.V. Polyakova, Ph.D. (chemistry), Institute of Inorganic Chemistry, SB RAS). Capillary electrophoresis is based on the migration and separation of cations under the action of an electric field due to their different electrophoretic mobility. The limits of detection for the quantification of various ions were 10^−3^–10^−4^% (federal nature conservancy procedural document 14.2:4.167–2000), and relative standard deviation (error) was ≤15%.

### 2.4. Statistical Analysis

Principal component analysis and 2B-PLS (2-block partial least squares) analysis [23] were performed using Statistica 8, Excel 16, and PAST 3.17 [24]. The 2B-PLS method is designed to compare two systems of traits or descriptions related to the same set of objects and is especially useful when there are many more variables than objects. In this case, for each system of descriptions, its own matrix of Euclidean distances between objects is built. This is always possible, for example, through nonmetric multidimensional scaling. For each distance matrix, principal components are calculated using the principal coordinate method [25]. Principal components are new traits of objects, but their number is always less than the number of objects. Next, two linear combinations of variables of each block (latent vectors, bicomponents) are calculated that most strongly (modulo) covary with each other. These bicomponents, each on its part, reflect the joint variation of both blocks the most because both variances of the two bicomponents and the correlation between them are reflected in the covariance. The process is repeated until the joint variation is exhausted. To interpret the obtained results, scatterplots of objects on the plane of paired bicomponents are useful, as are plots of correlations with paired bicomponents of any traits of the objects under consideration including those that were not included in the calculation of the bicomponents. Confidence intervals were computed via bootstrapping for the 2B-PLS methods, with N_boot_ = 2000 [26].

## 3. Results

### 3.1. Physicochemical Parameters of Lake Solenoe (Water, Bottom Sediments, and Floating Microbial Communities)

**Figure 3:**
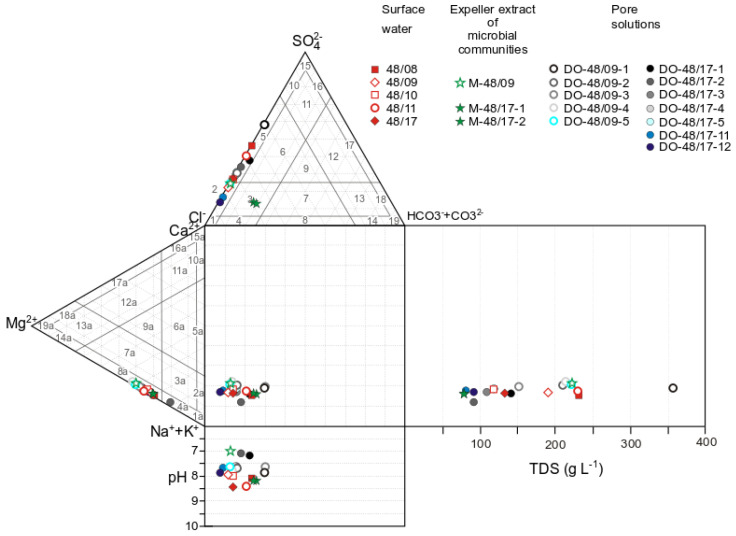
The Durov diagram for surface water, for the expeller extract of microbial communities, and for pore solutions of Lake Solenoe, in combination with the scheme of classification by the profile of the main anions Cl^−^, SO_4_^2^^−^, and HCO_3_^−^ and cations Na^+^+K^+^, Ca^2+^, and Mg^2+^ [27]. Anionic composition: 1: Cl, 2: Cl–(SO_4_), 3: Cl–(SO_4_)–(CO_3_), 4: Cl–(CO_3_): 5: Cl–SO_4_, 6: Cl–SO_4_–(CO_3_), 7: Cl–CO_3_–(SO_4_), 8: Cl–CO_3_, 9: Cl–SO_4_–CO_3_, 10: SO_4_–(Cl), 11: SO_4_–(CO_3_)–(Cl), 12: SO_4_–CO_3_–(Cl), 13: CO_3_–(SO_4_)–(CI), 14: CO_3_–(CI), 15: SO_4_, 16: SO_4_–(CO3), 17: SO_4_–CO_3_, 18: CO_3_–(SO_4_), and 19: CO_3_. Cationic composition: 1a: Na, 2a: Na–(Ca), 3a: Na–(Ca)–(Mg), 4a: Na–(Mg), 5a: Na–Ca, 6a: Na–Ca-(Mg), 7a: Na–Mg–(Ca), 8a: Na–Mg, 9a: Na–Ca-Mg, 10a: Ca–(Na), 11a: Ca–(Mg)–(Na), 12a: Ca–Mg–(Na), 13a: Mg–(Ca)–(Na), 14a: Mg–(Na), 15a: Ca, 16a: Ca–(Mg), 17a: Ca–Mg, 18a: Mg–(Ca), 19a: Mg.

The lake waters are classified as brines (salinity > 50 g L^−1^). Regardless of the salinity, Na^+^ is more abundant than Mg^2+^. The proportion of magnesium in (mol eqv) L^−1^ varies from 10% to 15%. Nonetheless, the ratios of Cl^−^ and SO_4_^2−^ (main anions) in the coastal zone change depending on weather conditions and, as a consequence, on the volume of the body of water and on solution salinity (Figure 3). The composition of the surface water of Lake Solenoe in cool and rainy years (2009, 2010, and 2017) was Na^+^–Mg^2+^–Cl^−^–(SO_4_^2−^), and in hot and dry years (2008 and 2011): Na^+^–Mg^2+^–Cl^−^–SO_4_^2−^. In the latter case, solution salinity increases from 120 to 230 g L^−1^. At a solution salinity of 230 g L^−1^, the highest SO_4_^2−^ content was registered (Figure 3).

**Figure 4:**
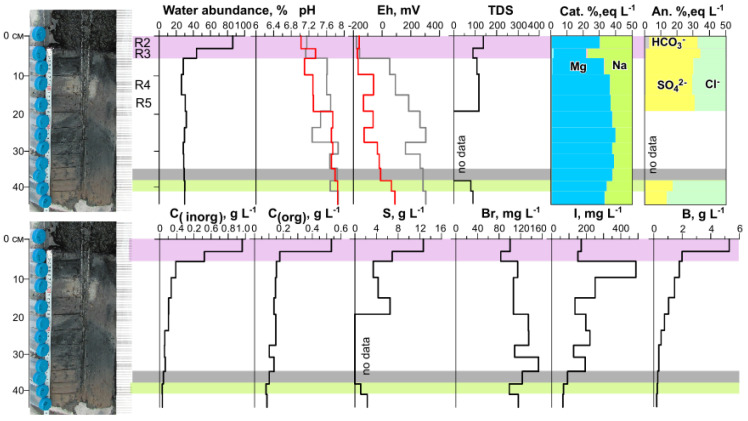
A change in water abundance, pH, Eh (red line: in suspension, gray: in a pore solution), salinity (total dissolved solids, g L^−1^), distribution of main cations and anions in a solution along the column depth, levels of inorganic (C_inorg_), organic carbon (C_org_), S, Br, I, and B in the pore solution of the sediment from Lake Solenoe according to 2017 data. Layers 1 and 2 of the sediment are highlighted in pink, they feature high water abundance and considerable C_org_ and C_inorg_ contents in the solution. The border of Eh change from (−) to (+) is marked in gray. The layer in which the main anions change from Cl^−^–SO_4_^2^^−^ to Cl^−^–(SO_4_^2^^−^) is highlighted in green.

At the beginning of the current research project, the pH and Eh of the pore solutions from bottom sediments were determined in a solution squeezed out of the material [14]. In 2010 and 2011, the changed Eh in the squeezed-out pore solutions was oxidizing (100–300 mV) [14]. Nonetheless, upper parts of the bottom sediments were enriched with organic matter, having a distinct smell of hydrogen sulfide. Determination of Eh in such media is a difficult task because when the equilibrium is disturbed, this parameter changes rapidly. To assess the actual conditions in the system, it is necessary to use various measurement techniques that involve isolation from the external environment [28,29]. To obtain more reliable data, a field experiment was conducted in 2017. The pH of the bottom sediments measured in suspension and the pH of the pore solution squeezed out in the laboratory were in good agreement with each other (Supplementary Table S1, Figure 4) and with the results of early studies [14].

The results of measuring Eh in the slurry (Eh^1^) and in the squeezed-out pore solution (Eh^2^) in the upper two most water-rich layers were very close to each other and indicated reducing conditions (Supplementary Table S1, Figure 4). In contrast, below 5 cm from the surface, Eh^1^ was found to be stably significantly lower than Eh^2^; values of the former were reducing, and those of the latter were oxidizing (Figure 4).

In 2017, when the samples were collected for the metagenomic analysis, the solution salinity in the coastal part (at the site where the floating microbial community developed) was 122 g L^−1^. The pH of the solution was 8.46, and Eh = 226 mV. The solution squeezed out of the microbial community differed from the surface solution of the lake in its lower salinity (75 g L^−1^) and a higher total and relative levels of HCO_3_^−^ (Supplementary Table S1). At the same time, C_org_ content proved to be comparable to that in the lake solution. In the main characteristics, the pore solutions were found to be similar to surface waters. Only the upper unconsolidated layer of the sediment, as noted earlier, was characterized by higher SO_4_^2−^, HCO_3_^−^, and C_org_ contents (Supplementary Table S1).

**Figure 5:**
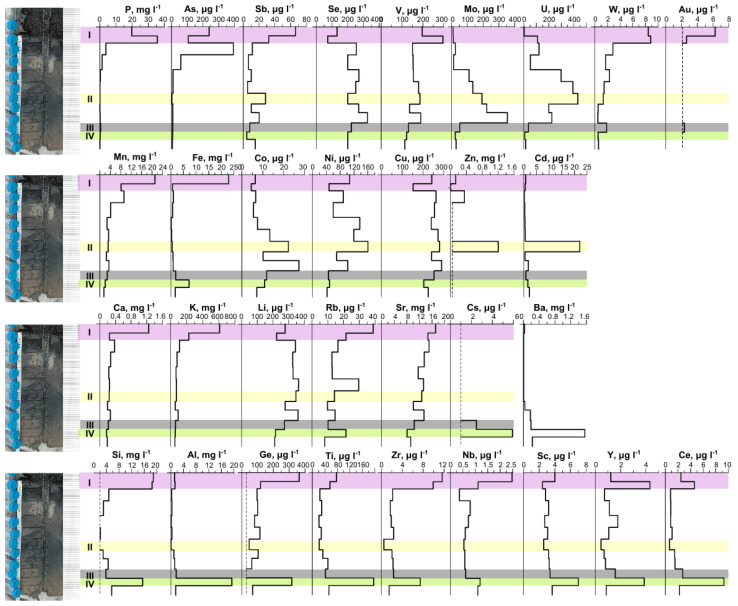
Distribution of elements in the pore solution of the sediment from Lake Solenoe according to 2017 data. (**I**) The layers of the sediment that are characterized by high water abundance and considerable concentrations of C_org_ and C_inorg_ in the solution are highlighted in pink. (**II**) The layer featuring the highest Zn and Cd contents is highlighted in yellow. (**III**) The border of Eh change from (−) to (+) is marked in gray. (**IV**) The layer in which the main anions change from Cl^−^-SO_4_^2^^−^ to Cl^−^-(SO_4_^2^^−^) is highlighted in green.

Initially, the concentration of trace elements was determined in a dried solution (salts) by synchrotron radiation X-ray fluorescence spectrometry. Already at this research stage, rather high levels of some elements were registered [14]. In 2017, for the first time, full trace element composition was investigated in detail in the surface solution, in the expeller extract from the floating microbial community, and in the pore solution from the bottom sediments (Supplementary Table S1, Figure 5). Unfortunately, the high salinity of the solution at present made it impossible to obtain reliable data on the concentration of some elements.

The composition of surface solutions (water) from Lake Solenoe, the expeller extract from the floating microbial community, and the pore solutions is presented in Supplementary Table S1.

The pore solutions also stood out in terms of high levels of elements, and along the cross-section of the bottom sediment, concentrations of most elements changed significantly. The layer in which the main anions changed was also distinguished by us in terms of its geochemical parameters. The levels of C_inorg_ and C_org_ in the pore solution from the upper layer of the sediment were the highest and decreased with depth. The highest iodine content was characteristic of the third layer and decreased with depth, whereas Br content along the column (cross-section) was relatively uniform. The upper two layers stood out as layers with distinguishing characteristics: the highest water abundance, highest concentrations of C_org_, C_inorg_, S, and other parameters (Figure 4). Evidently, the first and second layers differed from each other in many parameters, but at this research stage, they were separated into one system: the most active decomposition of organic matter.

The distribution of trace elements in the pore solution was not uniform along the cross-section (Figure 5). The amounts of C_inorg_, C_org_, and most trace elements in the upper unconsolidated layer of the bottom sediment were found to be the largest and to decrease sharply or gradually with depth (Figure 5). High P levels were identified in the pore solution from the upper 5 cm of the sediment.

### 3.2. Composition and Structure of Microbial Communities. Metagenomic Results

The metagenomic dataset comprised 398 million raw reads per sample with a length of 35–100 bp in samples R1 and R2; 139 million raw reads with length 35–150 bp in R3; 23 million raw reads with length 35–150 bp in R4; and 12 million raw reads with length 35–150 bp in R5.

In samples R1, R2, and R3, ~97% of the microbial community was bacteria in terms of the number of microbial cells (corresponding to sequence reads, i.e., genome copies), and 1.0–1.4% was archaea; and in samples R4 and R5, bacteria had relative abundance of 84% and 87%, respectively, and archaea 14% and 10%, respectively (Table 1). In the further analysis, eukaryotes were disregarded.

### 3.3. Taxonomic Profiling Based on 16S rRNA Sequences Obtained from Reads (phyloFlash)

**Figure 6:**
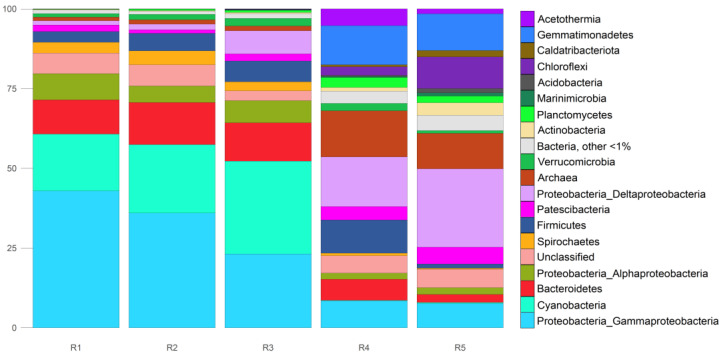
Taxonomic composition of the studied communities. Taxa according to WGS (16S RNA, phyloFlash, SILVA). R1: floating microbial community; R2–R5: bottom sediments.

According to the data obtained using the phyloFlash software (version 3.3b2), in the sample of the floating microbial community (R1), bacteria of the Proteobacteria phylum were predominant (52.4%), with the dominance of the class Gammaproteobacteria (42.9%) (Figure 6). Cyanobacteria took second place in terms of relative abundance (17.8%), and third place was taken by Bacteroidetes (10.7%) and Proteobacteria, class Alphaproteobacteria (8.2%). Spirochaetes and Firmicutes accounted for 3.4% each. All other bacterial phyla were scarce. In the floating microbial community, archaea represented 1.1%.

The sample of the unconsolidated layer of bottom sediment R2 was dominated by the same phyla of bacteria and archaea as in the floating microbial community. Proteobacteria constituted 42.9% (of them Gammaproteobacteria was 36%), Cyanobacteria 21.4%, and Bacteroidetes 13.2%. Minor phyla were Firmicutes (5.5%) and Spirochaetes (4.4%). Archaea had 1.4% relative abundance.

In the next layer of bottom sediment, R3, Proteobacteria were also the dominant bacteria (37.4% relative abundance; among them, Gammaproteobacteria, Deltaproteobacteria, and Alphaproteobacteria accounted for 23.2%, 7.2%, and 7.0%, respectively), as were Cyanobacteria (29.1%). In this layer, the abundance of Deltaproteobacteria increased significantly in comparison with the previous layer. Bacteroidetes were found to have a relative abundance of 12%. Superphylum Patescibacteria and phyla Verrucomicrobia, Spirochaetes, and Firmicutes represented 2% to 7% of all microbial cells here. The amount of detected archaeal sequences was still modest: 1.5%.

In the R4 layer of bottom sediments, located at a depth of 10–15 cm, relative abundance of archaea was sharply higher (up to 14.4%). In this layer, the major phyla of bacteria were Proteobacteria (26%, where Deltaproteobacteria accounted for 15.6%), Gemmatimonadetes (12.3%), and Firmicutes (10.4%). Acetothermia, Bacteroidetes, Chloroflexi, Patescibacteria, Planctomycetes, and Verrucomicrobia were found to be present at 2% to 9%.

In the next layer of bottom sediments, R5, the most abundant phyla of bacteria turned out to be Proteobacteria (34.3%, where Deltaproteobacteria accounted for 24.6%), then Gemmatimonadetes (11.5%) and Chloroflexi (9.9%). The relative abundance of archaea was still high (11.1% of all microbial cells). Patescibacteria, Actinobacteria, Bacteroidetes, Planctomycetes, and Caldatribacteriota accounted for 2% to 5.3%.

In all samples, there were sequences (3.1–6.6%) that could not be classified to good taxonomic depth, and only at the domain level.

**Figure 7:**
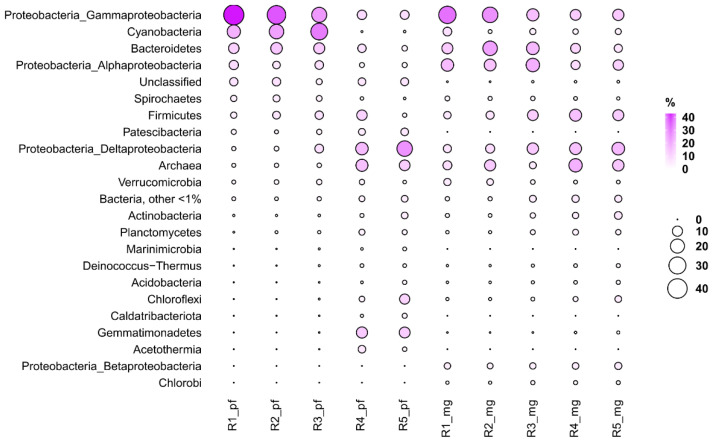
Abundant prokaryotic groups in Lake Solenoe. R_pf: Relative abundance of major taxa according to 16S rRNA reads in unassembled metagenomic datasets. R_mg: Relative abundance of the major taxa according to the analysis of all reads from the original dataset.

In general, in this study, MG-RAST data were consistent with the phyloFlash data. For example, in the studied samples, the proportion of sequence reads (meaning genome copies) from representatives of the Archaea domain was 1.1–14.4% and 4.4–17.9% according to the phyloFlash and MG-RAST data, respectively. The most abundant phyla were Euryarchaeota and Crenarchaeota. The highest relative abundance of archaea in both cases was found in the bottom sediment layer (R4) (Figure 7).

In the bacterial domain, the most abundant taxon was Proteobacteria, reaching 52.4% (phyloFlash) and 56.3% (MG-RAST) in the upper layer R1. The highest relative abundance of Alphaproteobacteria was registered in the first layer (phyloFlash) and in the third one (MG-RAST). In both cases, the highest relative abundance of Deltaproteobacteria was noted in the lowest layer, and the highest relative abundance of Gammaproteobacteria in the uppermost layer. According to both phyloFlash and MG-RAST, Bacteroidetes were most abundant in the upper layers with a maximum at R2. Cyanobacteria were abundant in upper layer R1 (MG-RAST) as well as in R2 and R3 (phyloFlash). According to phyloFlash and MG-RAST, Firmicutes were most abundant in the R4 layer, while Chloroflexi in R5.

The observed discrepancies were mainly related to variations in the abundance of representatives of some phyla and classes. In particular, a greater number of unclassified sequence reads was outputted by phyloFlash than by MG-RAST. There were also differences in candidate and/or small phyla. The taxonomic information obtained by extracting the sequences of the bacterial and archaeal 16S rRNA gene from metagenomic reads revealed approximately the same pattern of the community composition as that obtained by annotating all the reads from the original dataset.

### 3.4. Metabolic Features of the Microbial Communities

**Figure 8:**
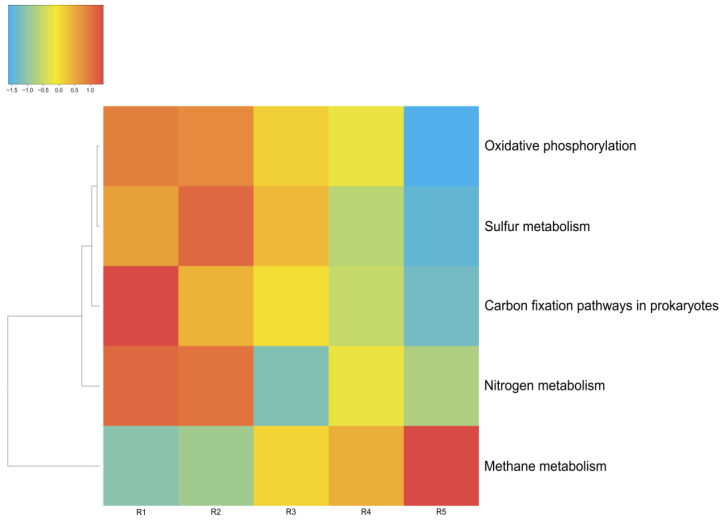
Energy metabolism in the microbial communities of the lake. The color range: −1.5 is the lowest relative abundance of genes involved in a metabolic process, and 1.0 is the highest relative abundance. Oxygenic and anoxygenic processes of photosynthesis were found in the microbial communities of the lake. Anoxygenic photosynthesis proceeds without oxygen emission; in this case, hydrogen and sulfides are most often electron donors. The fixation of carbon dioxide is carried out by microorganisms of all five of the analyzed layers.

The energy metabolism of the microorganisms in the microbial communities of Lake Solenoe was found to be mainly provided by the processes of oxidative phosphorylation (respiration). With increasing depth, the proportion of microorganisms living on oxidative phosphorylation decreases (Figure 8). As a replacement, mostly methane metabolism comes to the fore. Additionally, with increasing depth, the proportion of microbes that obtain energy via nitrogen respiration increases.

#### 3.4.1. Carbon Metabolism

**Figure 9:**
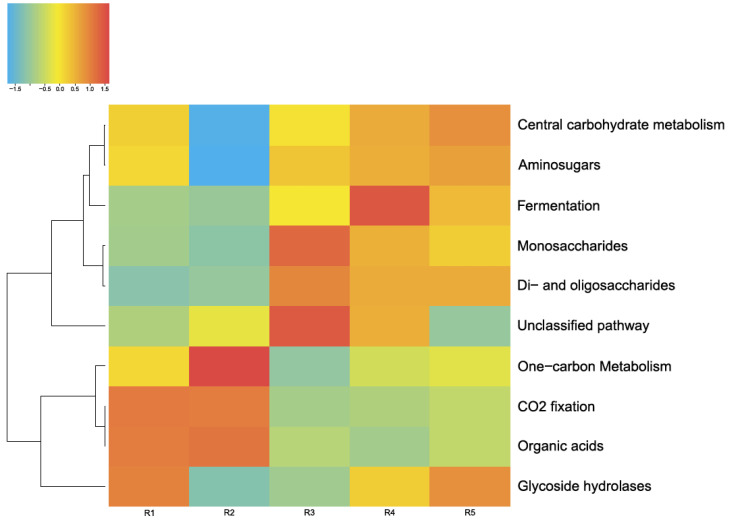
Pathways of carbon metabolism in the microbial communities of the lake. The color range: −1.5 is the lowest relative abundance of genes involved in a metabolic process, and 1.5 is the highest relative abundance.

Figure 9 shows the main pathways of carbon metabolism identified by MG-RAST tools in the five microbial communities studied by us in this lake. As depicted in Figure 8, the main way these microbial communities obtain carbon is carbohydrate metabolism (central carbohydrate metabolism). Furthermore, in all of these microbial communities, a network of metabolic reactions is present that involves the transfer of one-carbon groups from one compound to another (one-carbon metabolism) and carbon dioxide fixation. The latter phenomenon, coupled with oxygenic-photosynthesis processes, is characteristic of cyanobacteria, whose relative abundance in the studied communities is considerable.

In Lake Solenoe, aside from photosynthesis, benthic and deeper microbial communities are characterized by carbon fixation through methanogenesis and heterotrophic assimilation of carbon dioxide (Figure 8).

In the floating microbial community, archaea Halobacteriales and bacteria Alphaproteobacteria, Gammaproteobacteria, and to a lesser extent, other taxa are capable of fixing carbon in a nonphotosynthetic way. In benthic and deep communities, Deltaproteobacteria, Clostridia, Bacteroidetes, Bacilli, and to a lesser extent, other archaea and bacteria join the process.

Among the metagenomes in the lake in question, representatives of the orders Methanosarcinales and Methanococcales dominate among the methanogenic microorganisms. In the deep community, they are joined by Methanobacteriales, Methanomicrobiales, and other methanogenic archaea. Only methanogenic archaea of the order Methanosarcinales have a unique way of reducing carbon dioxide to methane: CO_2_ + 4H_2_ → CH_4_ + 2H_2_O

Archaea of the order Methanosarcinales, according to both phyloFlash and MG-RAST, predominated in the R3, R4, and R5 layers and were almost completely absent in the floating microbial community and in the surface layer on the bottom.

#### 3.4.2. Nitrogen Metabolism

Denitrification is an anaerobic process; therefore, it is not surprising that the key component of atmospheric-nitrogen fixation—iron protein NifH (nitrogenase)—predominates in sediment layers (R2 and R3). Nitrogenase NifH was not found here in the floating microbial community (Supplementary Figure S1). The floating microbial community probably carries out assimilatory nitrate reduction: nitrate => ammonia (i.e., (NO_3_)^−^ → (NO_2_)^−^ → NH_4_^+^) as well as denitrification: nitrate => nitrogen. These processes in the floating microbial community are mainly implemented by Proteobacteria (gamma and alpha) and Cyanobacteria.

In the other microbial communities, the spectrum of nitrate and nitrite reductases was broader due to additional enzymes including those characteristic of archaea, in good agreement with the observed greater relative abundance and greater species number of the archaea domain in the bottom layers. The greatest variety of denitrification enzymes was concentrated in the R3 layer of bottom sediments. Species of Alphaproteobacteria and Deltaproteobacteria dominated among denitrifiers, whereas archaea of the Halobacteria class and bacteria of the Flavobacteria, Cytophagia, and Chlorobia classes constituted a considerable proportion of such microbial cells. In deeper layers, there was a greater number of species of Clostridia and Actinobacteria capable of fixing inorganic nitrogen.

#### 3.4.3. Sulfur Metabolism

In all the tested layers and in the floating microbial community, SO_4_^2−^ was reduced to H_2_S via adenyl sulfate by sulfate adenylyltransferase [EC:2.7.7.4], phosphoadenosine phosphosulfate reductase [EC:1.8.4.8], and adenylylsulfate kinase [EC:2.7.1.25] in the following reactions: ATP + sulfate → diphosphate + adenylyl sulfate [RN:R00529], thioredoxin + 3’-phosphoadenylyl sulfate ↔ thioredoxin disulfide + sulfite + adenosine 3’,5’-bisphosphate (Supplementary Figure S2).

Sulfur metabolism was most pronounced in layers R2 and R3, where the oxidation of organic substrates also containing sulfur took place in addition to the chemosynthesis processes. In the process of assimilatory sulfate reduction, sulfate is reduced, and then the sulfur is incorporated into proteins.

The sulfur cycle is a complex network of chemical reactions that can either occur spontaneously in the environment, depending on temperature and pH, or can be controlled by microbial enzymatic systems. The recovery of sulfur compounds is a typical process catalyzed by microorganisms in hypersaline lakes [30].

In the floating microbial community, the processes of sulfur metabolism are implemented by bacteria, while in the other microbial communities here, archaea also participate in the sulfur metabolism, and their relative abundance increases with depth. The greatest diversity of sulfur metabolism enzymes was concentrated in the R3 layer, where 512 species-specific enzymes, mainly bacterial, were identified. In the deeper layers, there were fewer bacterial enzymes, and were joined by sulfur metabolism enzymes characteristic of methanogenic archaea.

## 4. Discussion

This paper presents the results of a comprehensive study on the microbial profile of Lake Solenoe: from assessing the taxonomic composition to analyzing the functions of the microbial community. The presented taxonomic profiling based on recovered 16S rRNA data from unassembled metagenomic data (phyloFlash) and based on the analysis of all reads from the original dataset (MG-RAST) made it possible to obtain results [16] elucidating the roles of various taxonomic groups in the structure of the microbial communities under study.

In addition, using principal component analysis, a significant direct positive correlation was revealed between the data obtained via phyloFlash and those obtained via MG-RAST (r = 0.97, *p* < 0.05). Consequently, both methods are applicable to describing and analyzing taxonomic composition of a microbial community.

In this study, analyses of the microbial-community composition, metabolic pathways, and geochemical data uncovered substantial changes along the vertical axis: from surface floating microbial community R1 to deep layer R5 of the bottom sediment. It was revealed that the lower sediment layers R4 and R5 were in the zone of stability for most of the geochemical parameters (pH, TDS, Mg, Na, SO_4_, Cl, C_org_, C_inorg_, Br, P, and others), and the changes were significant for Eh, S, I, As, Mo, U, Ni, and some other parameters (Figure 4 and Figure 5, Supplementary Table S1). On the other hand, a significant bifurcation of many parameters occurred in layers R2 and R3 (e.g., TDS, Mg, Na, Cl, C_org_, C_ingor_, S, Br, B, P, As, and Sb), while Eh, I, main anions, Mo, W, Co, Cd, Ba, Si, and Al remain fairly stable.

It should be noted that, in comparison with seawater, which is parental water for the saline lakes of the Barabinskaya steppe, the concentration of some trace elements was still at the same level or comparable (Li, B, Si, Br, and I), but for the majority, the level was much higher in the lake. It was found here that levels of many trace elements (Sc, Se, Zr, Nb, Mo, Ag, Cd, Sn, Hf, Ta, Pb, Th, U and the rare-earth elements [REEs]) in the surface water, in the expeller extract from the microbial community, and in the pore solution from the bottom sediment were 2-, 3-, 4-, and even 5-orders of magnitude higher than those in modern seawater (Figure 5, Supplementary Table S1). The concentration of trace elements in the expeller extract from the microbial community was higher than that in the surface water, especially for Mn, Fe, Co, Zn, As, Sb, Y, REEs, Pb, and Th.

Here Fe, U, and Mo showed a distribution typical of organic matter containing marine sediments [31]. It is believed that the distribution of Fe and U in the pore solution and sediment reflects the change from oxidizing to reducing conditions and determines the depth of the redox-cline [32]. In the pore solution, the peaks of the highest Fe and U levels did not match. It is known that Fe(III) under reducing conditions is converted to more soluble Fe(II), and this process is controlled by the depth of O_2_ penetration [31]. Uranium, in contrast, is soluble in the presence of O_2_ and is in the form of UO_2_^2+^ (uranyl ion); in a reducing environment, it precipitates as UO_2_ [33]. According to these data, the redox-cline is located in the sediment of Lake Solenoe at a depth of 35–38 cm [34]. On the other hand, such a distribution of elements in the lake solution is also explained by the activity of sulfate-reducing microorganisms [35], many of which determine the oxidation and/or reduction reactions of elements with variable valence including U, Fe, Mn, and Mo [36]. A similar distribution of U and Fe is also seen in organic matter-rich pore solutions of small freshwater lakes in Siberia [37]. There is evidence of catalytic reduction of U (VI) by means of Fe(II) compounds [38]. In real-world sediments, there is most likely a cumulative contribution of different processes. The data obtained by the present authors show that Eh increased with depth, and the highest U concentration in the pore solution corresponded to more reducing conditions.

In recent years, there have been reports on the active complexation of lanthanides and their transport with humic acids and other organic ligands and REEs transport [39,40]. Research confirms that complexation with organic ligands plays a huge role in the fractionation of REEs in natural, organic matter-rich riverine and underground waters [41,42]. Perhaps this explains the high levels of many metals in the pore solution from the upper layer of bottom sediments in the lake; this solution is enriched with dissolved and colloidal organic matter.

**Figure 10:**
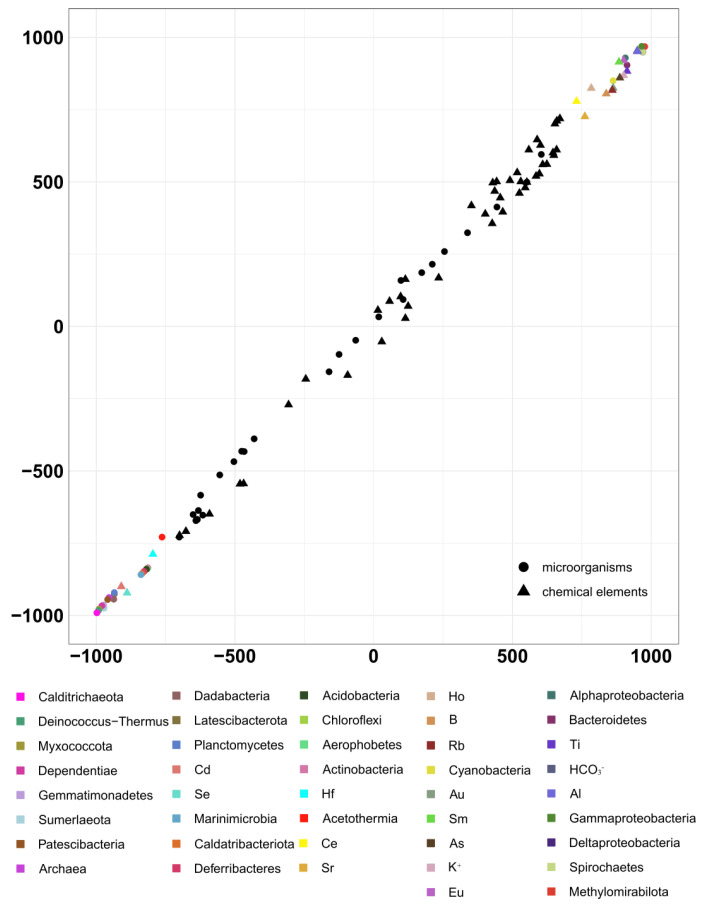
Results from the analysis of the second pair of datasets by the 2B-PLS method. Results on taxonomic composition (according to phyloFlash) and the geochemical factors potentially affecting it. B1: Axis 1: geochemical datasets, B2: Axis 1: taxonomical datasets.

A significant direct positive correlation was revealed between the presented geochemical and taxonomic datasets, and the correlation coefficient was 0.99 (*p* < 0.05; Figure 10).

Consequently, in the analyzed ecosystem of Lake Solenoe, activities of microorganisms can substantially affect the distribution of elements from the surface to deep sediment layers. The microbial activities are also probably the reason for the high solubility of the elements that migrate only slowly in neutral and alkaline solutions.

The observed changes in the geochemical components lead to alterations in the composition of the microbial communities, and conversely, the processes taking place in these communities are so active that they cause changes in some geochemical components. That is, this is a united system of mutually influencing and complementary factors.

For instance, in the analyzed layers, with increasing depth, there was an increase in the relative abundance of archaea, which can play an ecological role as the main decomposers of organic matter in soda and saline lakes [13]. Their relative abundance was very sharply higher in the fourth layer (R4), probably due to changes in some geochemical parameters that occurred in the upper layers (Figure 4 and Figure 5, Supplementary Table S1). The higher relative abundance of Euryarchaeota is probably also explained by their involvement in methanogenesis [43], consistent with the data on methane metabolism. Namely, it was the lower layers where the largest number of methane metabolism enzymes was noted. The fourth layer manifested uniqueness (perhaps because of the absence of the third layer, there was no smooth transition in the distribution of these numbers, and a sharp increase could be seen), but here, not only did the abundance of archaea increased most sharply and to the highest level, but so did the number of bacterial phyla, for example, Acetothermia and other phyla that were very scarce in the upper layers. Here, due to the absence of light, the abundance of Cyanobacteria was sharply lower. In the R4 layer, the set of dominant taxa was different: Deltaproteobacteria and Gemmatimonadetes were now predominant among bacteria, while Euryarchaeota were now dominant among the archaea. Additionally, in this layer, relative abundance of Firmicutes reached its maximum.

To sum up, in layers R2 and R3, there was a noticeable inflexion point of the geochemical parameters, followed by an alteration in the structure and composition of the microbial community, leading to changes in the community’s metabolism and functions, which in turn could cause biogenic mineral formation and other processes. For example, it is known that many members of Bacteroidetes can consume biopolymers such as chitin, cellulose, xylan, and proteins and are therefore often regarded as a key taxon for the remineralization of organic matter in a marine environment (e.g., [43]).

In the current work, the examination of the metabolic pathways present in the microbial communities of the lake and the performed geochemical analysis allowed us to distinguish three ecological niches with implemented geochemical processes characteristic for each group of microorganisms.

The first one was the floating microbial community (R1), in which cyanobacteria made up the main contribution to the formation of organic matter. Similar processes occurred almost everywhere, for example, in the microbial communities of the alkaline aquatic ecosystems of the Baikal region [44]. In terms of carbon cycling, soda lakes are among the most productive natural ecosystems in the world because they actively absorb atmospheric carbon but also contribute significantly to the production and emissions of greenhouse gases [45,46]. The phylum Cyanobacteria plays an important part in the geochemistry of Lake Solenoe. The photosynthesis processes performed by cyanobacteria drive a sharp increase in pH as a consequence of the consumption of CO_2_ and HCO_3_^−^ and, as a result, an increase in CO_3_^2−^ levels [47]. A shift of pH can contribute to a change in the solubility and migration of substances from the bottom sediments of the lake, thereby influencing the biogeochemical cycle of some elements.

The second niche was the upper layers of the bottom sediment (R2 and R3). Here, the floating microbial community and allochthonous organic matter settling to the bottom were destroyed. The organic mass of the *Artemia* crustacean was also processed here. Sulfur metabolism was most pronounced in these layers of bottom sediments. In this study, in the deeper layers that were categorized into the third ecological niche, archaea participated in sulfur metabolism. For all communities, the proportion of inorganic-sulfur assimilation enzymes significantly exceeded the proportion of organic-sulfur assimilation enzymes. Sulfates were reduced to sulfites, molecular sulfur, and hydrogen sulfide.

In the ecosystem of Lake Solenoe, a heterogeneous sulfur cycle under haloalkaline conditions was identified. The obtained biogeochemical profiling combined with the metagenomic analysis revealed unique communities of sulfur-oxidizing bacteria in layers R2 and R3. An elevated activity of sulfur-metabolizing bacteria was also present in the bottom layer of Cock Soda Lake (Kulunda Steppe, southwestern Siberia, Russia) [48]. The peak of microbial activity coincided with a sharp drop in oxygen concentration and in redox potential. Inorganic sulfur compounds can serve as energy sources for prokaryotes and are important substrates for microbial growth in general. The processes of sulfur assimilation play a major role in the microbial communities of this lake. In surface and pore solutions, the concentration of oxidized sulfur as part of the SO_4_^2−^ anion is high. Redox reactions of sulfur are a source of energy in chemosynthesis. In all of the tested layers and in the floating microbial community, SO_4_^2−^ was reduced to H_2_S via adenyl sulfate by sulfate adenylyltransferase [EC:2.7.7.4], phosphoadenosine phosphosulfate reductase [EC:1.8.4.8], and adenylylsulfate kinase [EC:2.7.1.25] in the following reactions: ATP + sulfate → diphosphate + adenylyl sulfate [RN:R00529], and thioredoxin + 3’-phosphoadenylyl sulfate ↔ thioredoxin disulfide + sulfite + adenosine 3’,5’-bisphosphate.

Representatives of the genus *Desulfovibrio* are resistant to heavy metals and are able to reduce compounds of chromium(III), copper(II), manganese(II), nickel(II), and zinc(II). Representatives of Deltaproteobacteria oxidize organic compounds to water and CO_2_.

In the metagenomes of Lake Solenoe, both methanotrophic processes (aerobic oxidation of CH_4_) and methanogenesis were detected. It is known that methanotrophs are responsible for considerable consumption of CH_4_ in saline and alkaline lakes [49,50] and that methanogenesis is likely more rapid in the sediments of the lake under study. In methanogenesis, the Wood–Ljungdahl (WL) pathway (i.e., the acetyl–CoA pathway) allows microorganisms to use hydrogen as an electron donor and to employ carbon dioxide as an electron acceptor and a building block for the biosynthesis of organic molecules [51]. The WL pathway is one of the most important metabolic cascades for energy production and carbon fixation [52]. When using the WL pathway for energy production and carbon fixation, most bacteria generate acetate as a final product (acetogens), whereas most archaea produce methane (CO_2_-reducing methanogens) [53]. Due to the high sensitivity of WL pathway enzymes to oxygen, this pathway occurs only among obligate anaerobes (methanogenic archaea, clostridia, and some sulfate-reducing bacteria) [54,55]. In extremely saline habitats, methylotrophic methanogenesis, implemented by facultative methylotrophs, is considered the dominant metabolic pathway. These microorganisms metabolize one-carbon organic compounds: formate, methanol, methylamines, and bicarbon acetate. In layers R3, R4, and R5, there were microbes capable of synthesizing 3-hydroxypropionate from CO_2_ via the Fuchs–Holo cycle [56].

The enzymes in the pathways of nitrogen metabolism contain ions of molybdenum, cobalt, iron, nickel, sulfur, or other elements, whose concentration was high in the expeller extract of the floating microbial community and was quite high in the pore solutions from the bottom sediments. In such complexes, the nitrogen molecule is polarized in a field of a metal atom and is attached to the metal atom via one nitrogen atom; as a result, the frequency of stretching vibrations in the nitrogen molecule changes and its reactivity increases. In “nitrate respiration,” nitrates and nitrites serve as the final acceptor of electrons. A substantial proportion of the bottom microbial communities of this lake belongs to microorganisms that practice sulfur assimilation. In “sulfate respiration,” sulfates serve as the final acceptor of electrons. Nitrates are reduced to molecular nitrogen or ammonia, whereas sulfates are reduced to sulfites, molecular sulfur, and hydrogen sulfide. Iron and sulfur-containing ferredoxins act as the primary carrier of electrons in nitrate and sulfate respiration.

**Figure 11:**
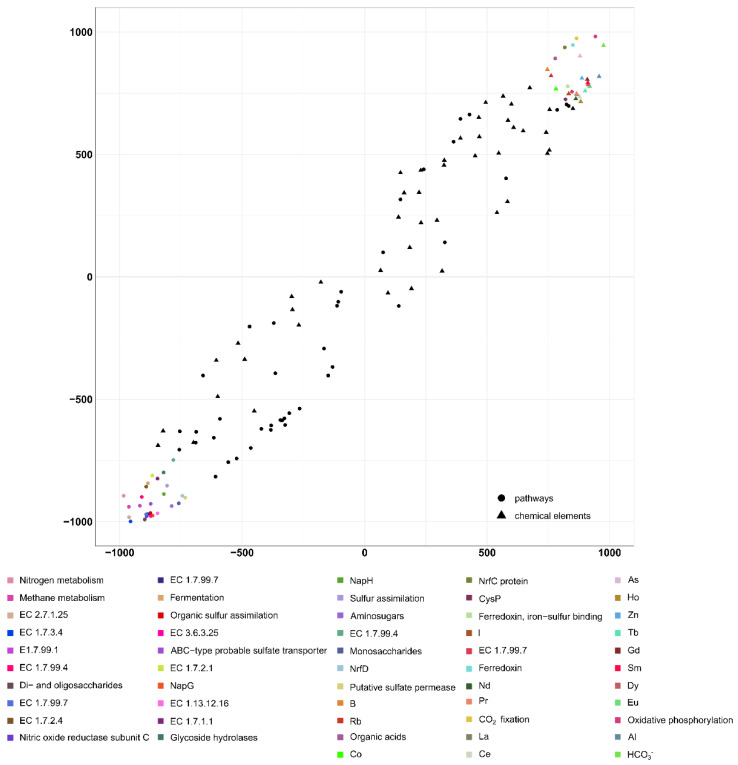
Results from the analysis of the second pair of datasets by the 2B-PLS method. Results on the metabolic pathways and the geochemical factors potentially affecting them. B1: Axis 1: geochemical datasets, B2: Axis 1: metabolic pathways.

A significant direct positive correlation was found here between the geochemical parameters and metabolic processes: the correlation coefficient was 0.95 (*p* < 0.05; Figure 11).

The presented results describe the entire spectrum of prokaryotes that developed in the floating microbial community, in the upper layer of silt, and in the bottom sediments of Lake Solenoe during the study period, thereby taking a snapshot of the microbiome in this ecosystem; these findings may be a starting point for further in-depth studies.

In this work, the patterns of change in the composition and structure of microbial communities were identified for the first time in a saline lake along the vertical axis, as were specific features of their metabolism depending on geochemical conditions. Nonetheless, much remains to be elucidated about the reverse relation: for example, the impact of the microbial community on geochemical gradients, the influence of microbial activity and metabolic processes on the concentrations of certain elements in the environment, and the possibility of their accumulation by the microbial community in vivo or postmortem. In geochemical and biogeochemical studies on the nature of metal-bearing carbonaceous deposits, two alternative points of view are currently dominant: (1) concentration of U, Mo, V, Ni, Au, Ag, and platinum group elements by living bacterial communities, and (2) sorptional accumulation of microelements by products of microbial decomposition. The present work shows that in the “saline lake (basin) water—bacterial community—sediment” system, there is an association of bacterial communities existing in different physicochemical circumstances, and this collaboration sequentially transforms the dissolved form of elements into sorptional, biomineral, and mineral forms.

Thus, the identified patterns suggest that microorganisms play a leading role in ecological homeostasis and in the geochemical processes taking place in the ecosystem of the analyzed saline lake. The detected physiological activities of microorganisms such as the ability to accumulate rare elements and biogenic synthesis of inorganic compounds, undoubtedly require further research, cloning of specific enzymes, or the isolation of microorganisms with promising biotechnological properties. More detailed spatial or temporal sampling in the future may help us to understand in more detail the influence of environmental gradients (e.g., of salinity and geochemical composition as well as gradients of other parameters such as pH and temperature) on specific microbial populations and functional microbial groups. Combining transcriptomic and/or proteomic approaches with in situ techniques is being planned.

## 5. Conclusions

The metagenomic approach used in this work helped to determine the composition and structure of the floating microbial community, the upper layer of silt, and the strata of bottom sediments in a natural hypersaline lake located in the south of western Siberia (Novosibirsk Oblast, Russia) by two bioinformatic methods.

Metabolic pathways were reconstructed to investigate the metabolic potential of microbial communities in terms of organic-carbon remineralization in soda brines and regarding other presumed roles of these communities in the biogeochemical cycle. Relationships between different taxa of microorganisms and their potential participation in biogeochemical transformations of C, N, and S were identified in a comparative structural analysis that included various ecological niches.

Research on taxonomic and functional profiles of a wider range of communities in this lake and other lake systems is being planned including their seasonal fluctuations and the stratification of the vertical profile of the lakes. In conclusion, the present study offers a more complete overview of the biogeochemical cycles associated with the activities of microbial communities in this and other lake ecosystems.

## Figures and Tables

**Table 1:** Proportions (relative abundance, %) of archaea, bacteria, and eukaryotes in the microbial communities of the studied samples.

Domain	Layer				
	R1	R2	R3	R4	R5
Archaea	1.08	1.32	1.43	14.17	10.72
Bacteria	97.19	97.25	96.48	84.67	87.28
Eukaryota	1.73	1.43	2.09	1.16	2.00

## Data Availability

Not applicable.

## Supplementary Materials

The following supporting information can be downloaded at: https://www.mdpi.com/article/10.3390/biology11040605/s1, Figure S1: Pathways of nitrogen metabolism in the microbial communities of the lake; Figure S2: Pathways of sulfur metabolism in the microbial communities of the lake. Table S1: The geochemical composition of Lake Solenoe, the expeller extract from the floating microbial community, and the pore solutions from bottom sediments.
