# Supplementary material for: Diversity and Metabolism of Microbial Communities in a Hypersaline Lake along a Geochemical Gradient
Source: Biology (Basel). 2022 Apr 15;11(4):605. doi: 10.3390/biology11040605 (PMC9031644; doi:10.3390/biology11040605)
Supplement: Supplementary file 1 [file biology-11-00605-s001.zip › biology-1604054-supplementary.pdf]

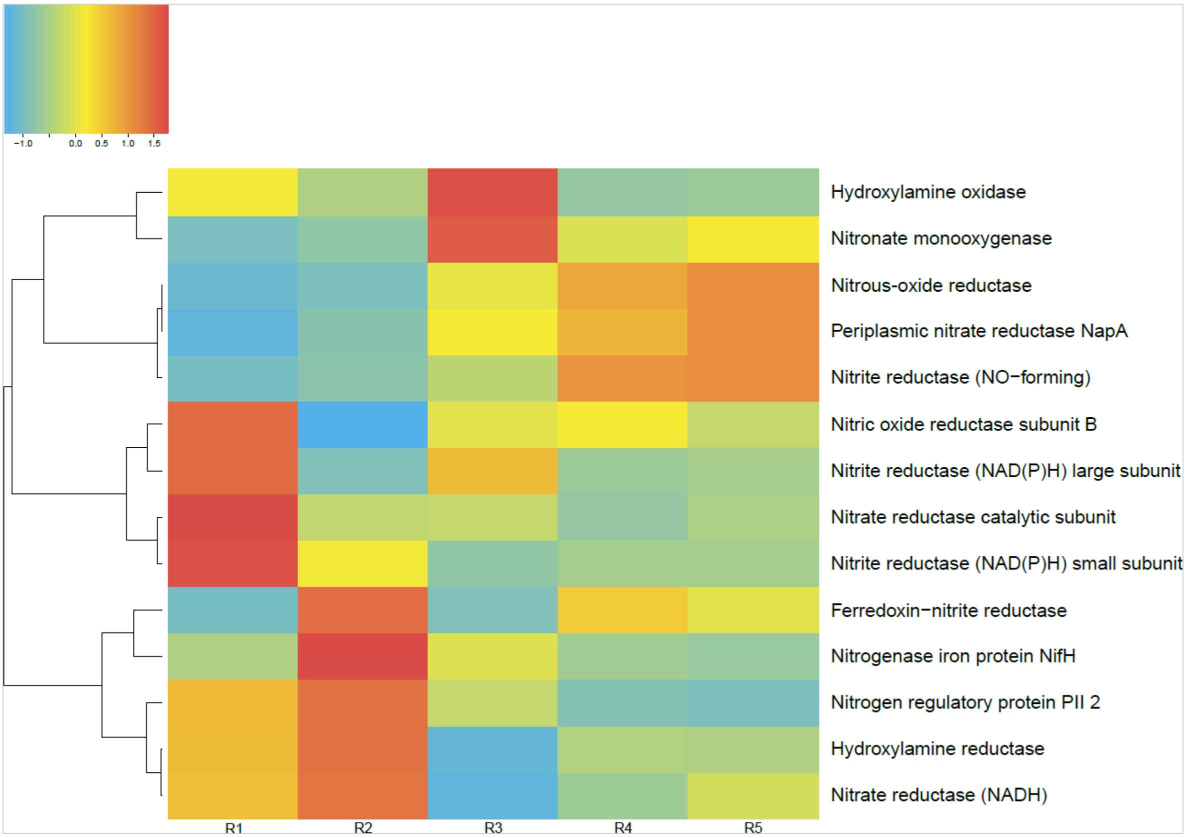

Figure S1. Pathways of nitrogen metabolism in the microbial communities of the lake.

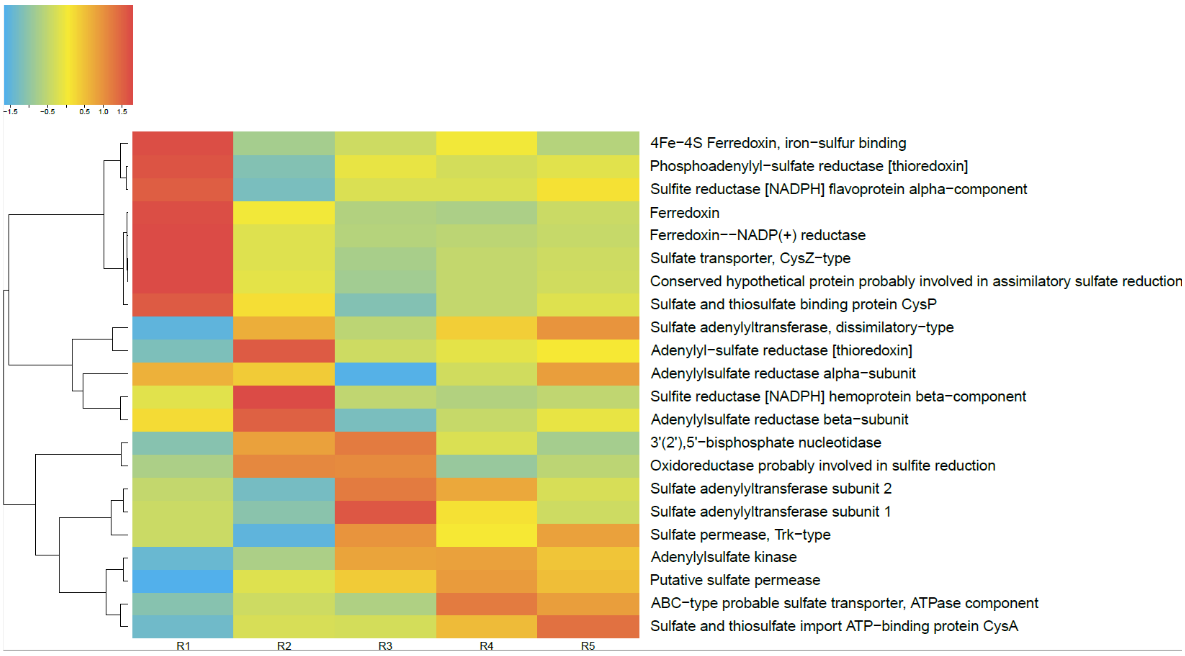

Figure S2. Pathways of sulfur metabolism in the microbial communities of the lake.

Table S1. The geochemical composition of Lake Solenoe, of the expeller extract from the floating microbial community, and of the pore solutions from bottom sediments.

Parameter	Unit	Layer				
		**R1**	**R2**	**R3**	**R4**	**R5**
**Ca^{2+}**	mg L^{-1}	345	530	245	277	228
Mg^{2+}	mg L^{-1}	2070	9160	3950	7910	7860
Na^{+}	mg L^{-1}	8850	39300	28200	26400	25600
K^{+}	mg L^{-1}	180	270	226	78	67
HCO^{3-}	mg L^{-1}	6110	4890	2660	691	539
DOC	mg L^{-1}	170	532	175	145	134
SO_4^{2-}	mg L^{-1}	3700	35000	20700	12900	19500
Cl^{-}	mg L^{-1}	14200	52600	43400	53200	52300
TDS	g L^{-1}	75	142	100	102	106
pH	pH	8.21	7.02	7.35	7.10	7.29
Eh^1	mV	263	-160	-172	-60	-130
Eh^2	mV	-	-180	-158	93	187
Li	µg L^{-1}	347	278	223	326	328
B	µg L^{-1}	3650	5300	1990	1460	1040
Al	µg L^{-1}	1220	1190	1330	373	130
Si	µg L^{-1}	3700	18900	18500	3080	<2000
P	µg L^{-1}	4560	19800	35700	1360	<3000
Sc	µg L^{-1}	2.54	4.00	2.42	3.21	2.70
Ti	µg L^{-1}	54	77	56	30	21
V	µg L^{-1}	174	197	296	153	155
Cr	µg L^{-1}	64	99	53	66	70
Mn	µg L^{-1}	4500	21100	8120	4100	2700
Fe	µg L^{-1}	2400	22900	623	696	105
Co	µg L^{-1}	8.1	6.4	4.4	5.5	7.4
Ni	µg L^{-1}	24	108	48	60	137
Cu	µg L^{-1}	303	243	153	256	240
Zn	µg L^{-1}	1860	133	<10	<48	<48
Ga	µg L^{-1}	<1	7.5	7.5	<1	<1
Ge	µg L^{-1}	61	364	119	100	81
As	µg L^{-1}	155	239	107	59	9
Se	µg L^{-1}	<240	133	73	200	270
Br	mg L^{-1}	124	100	83	107	107
Rb	µg L^{-1}	26	39	22	13	13
Sr	µg L^{-1}	14000	17100	14700	13500	11600
Y	µg L^{-1}	3.17	1.2	4.3	1.1	1.8
Zr	µg L^{-1}	3	12	9.9	1.7	1.9
Nb	µg L^{-1}	1	2.5	1.1	0.8	0.7
Mo	µg L^{-1}	4.9	9.7	11.1	12.7	111.3
Cd	µg L^{-1}	0.19	<1	<1	0.38	0.50
Sb	µg L^{-1}	11.4	66	32.4	6.7	10.2
I	µg L^{-1}	499	171	152	252	135
Ba	µg L^{-1}	<15	30	<15	< 15	< 15
La	µg L^{-1}	3.34	0.88	1.64	0.46	0.78
Ce	µg L^{-1}	7.91	1.90	4.62	0.82	0.84
Pr	µg L^{-1}	0.89	0.26	0.42	0.15	0.25
Nd	µg L^{-1}	3.55	0.67	1.75	0.32	0.60
Sm	µg L^{-1}	0.53	0.31	0.37	bdl	bdl

Eu	μg L^{-1}	0.17	0.12	0.16	bdl	bdl
Gd	μg L^{-1}	0.83	0.40	0.40	bdl	bdl
Tb	μg L^{-1}	0.18	0.07	0.11	bdl	bdl
Dy	μg L^{-1}	0.69	0.40	0.52	bdl	bdl
Ho	μg L^{-1}	0.17	0.07	0.14	bdl	bdl
Er	μg L^{-1}	0.34	0.14	0.53	bdl	bdl
Tm	μg L^{-1}	0.07	0.04	0.15	bdl	bdl
Yb	μg L^{-1}	0.25	0.14	0.58	bdl	bdl
Lu	μg L^{-1}	0.008	0.02	0.06	bdl	bdl
Hf	μg L^{-1}	0.14	0.2	<0.001	0.51	0.37
Ta	μg L^{-1}	1.38	0.6	0.22	0.82	0.75
W	μg L^{-1}	3.3	8.5	8.9	1.7	2.3
Au	μg L^{-1}	4.2	6.3	2.63	<2.1	<2.1
Pb	μg L^{-1}	18	<10	<10	14	14
Th	μg L^{-1}	7.4	1.3	2.4	1.2	1.2
U	μg L^{-1}	13	2	110	53	298

Note. [Be] < 5 μg L^{-1}, [Cs] < 1 μg L^{-1}, [Tl] < 1 μg L^{-1}. bdl: below detection limit. TDS: total dissolved solids.
